# Supplementary material for: mTORC1 couples cyst(e)ine availability with GPX4 protein synthesis and ferroptosis regulation
Source: Nat Commun. 2021 Mar 11;12:1589. doi: 10.1038/s41467-021-21841-w (PMC7952727; doi:10.1038/s41467-021-21841-w)

Supplementary Information

mTORC1 couples cyst(e)ine availability with GPX4 protein synthesis and ferroptosis regulation

Yilei Zhang[1], Robert V. Swanda[2], Litong Nie[1], Xiaoguang Liu[1], Chao Wang[1], Hyemin Lee[1], Guang Lei[1], Chao Mao[1], Pranavi Koppula[1, 3], Weijie Cheng[1], Jie Zhang[1], Zhenna Xiao[4], Li Zhuang[1], Bingliang Fang[5], Junjie Chen[1], Shu-Bing Qian[2, 6], Boyi Gan[1, 3 *]

[1] Department of Experimental Radiation Oncology, the University of Texas MD Anderson Cancer Center, Houston, TX 77030, USA.

[2] Graduate Field of Biomedical and Biological Sciences, Cornell University, Ithaca, NY 14853, USA.

[3] The University of Texas MD Anderson UTHealth Graduate School of Biomedical Sciences, Houston, Texas 77030, USA.

[4] Department of Genetics, the University of Texas MD Anderson Cancer Center, Houston, TX 77030, USA.

[5] Department of Thoracic and Cardiovascular Surgery, the University of Texas MD Anderson Cancer Center, Houston, TX 77030, USA.

[6] Division of Nutritional Sciences, Cornell University, Ithaca, NY 14853, USA.

Keywords: ferroptosis, cysteine, cystine, GPX4, mTORC1, protein synthesis, cancer treatment

* Corresponding Author: Boyi Gan. E-mail: bgan@mdanderson.org; Phone: 713-792-8653.

Supplementary Figure 1

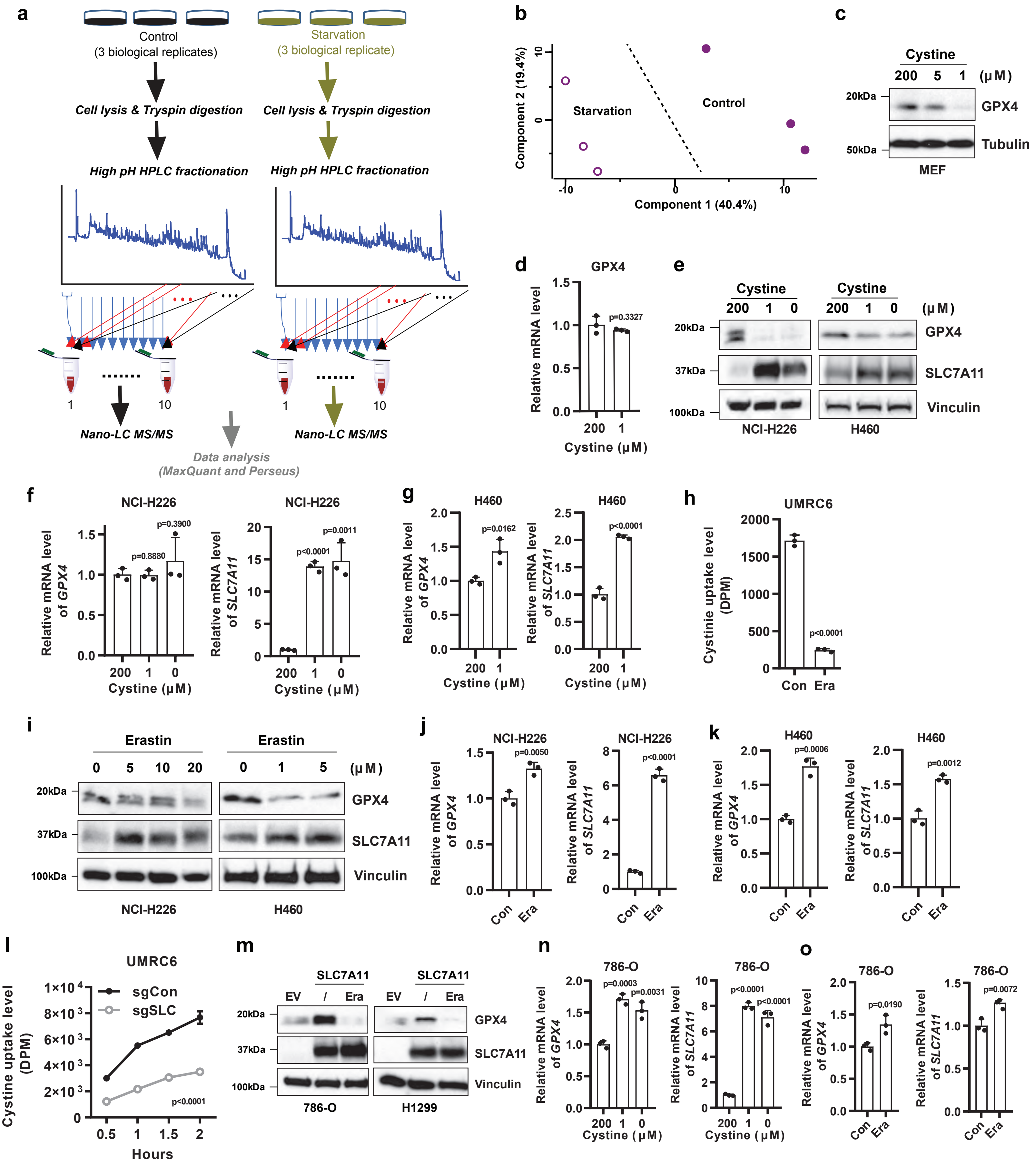

Supplementary Figure 1. SLC7A11-mediated cystine uptake promotes GPX4 protein expression.

a The flowchart of experimental design for proteomic studies. **b** Samples in triplicate were separated into 2 parts (Treatment and Control) using 2-dimensional principal component analyses. **c** Mouse Embryonic Fibroblast (MEF) cells were cultured in media with indicated concentration of cystine for 20 hours. Protein levels were evaluated by Western blotting. **d** *GPX4* gene mRNA level was measured by RT-PCR in MEFs cultured in media with indicated concentration of cystine for 20 hours. n=3. **e** NCI-H226 and H460 cells were cultured in media with indicated concentration of cystine for 24 hours. GPX4 and SLC7A11 protein levels were evaluated by Western blotting. **f-g** *GPX4* and *SLC7A11* gene mRNA levels were measured by RT-PCR in NCI-H226 (**f**) and H460 (**g**) cells treated as described in **e**. n=3. **h** Cystine uptake levels measured in UMRC6 cells treated with or without 10 μM erastin for 20 hours. n=3. **i** NCI-H226 and H460 cells were cultured in media with indicated concentration of erastin for 24 hours. GPX4 and SLC7A11 protein levels were evaluated by Western blotting. **j** *GPX4* and *SLC7A11* gene mRNA levels were measured by RT-PCR in NCI-H226 cells treated with 20 μM erastin for 24 hours. n=3. **k** *GPX4* and *SLC7A11* gene mRNA levels were measured by RT-PCR in H460 cells treated with 5 μM erastin for 24 hours. n=3. **l** Cystine uptake levels measured in control (sgCon) and SLC7A11-knockout (sgSLC) cells at indicated time points. n=3. **m** SLC7A11-overexpressed 786-0 or H1299 cells were treated with or without 10 μM erastin for 24 hours. Protein levels were evaluated by Western blotting in indicated cells. **n** *GPX4* and *SLC7A11* gene mRNA levels were measured by RT-PCR in 786-O cells cultured in media with indicated concentration of cystine for 20 hours. n=3. **o** *GPX4* and *SLC7A11* gene mRNA levels were measured by RT-PCR in 786-O cells treated with 5 μM erastin for 20 hours. n=3. For all panels, error bars are mean ± SD. n indicates biologically independent repeats. *P* value was determined by two-tailed unpaired Student's t-test. For panel **l**, *p* value was determined by two-way ANOVA test. Source data are provided as a Source Data file.

Supplementary Figure 2

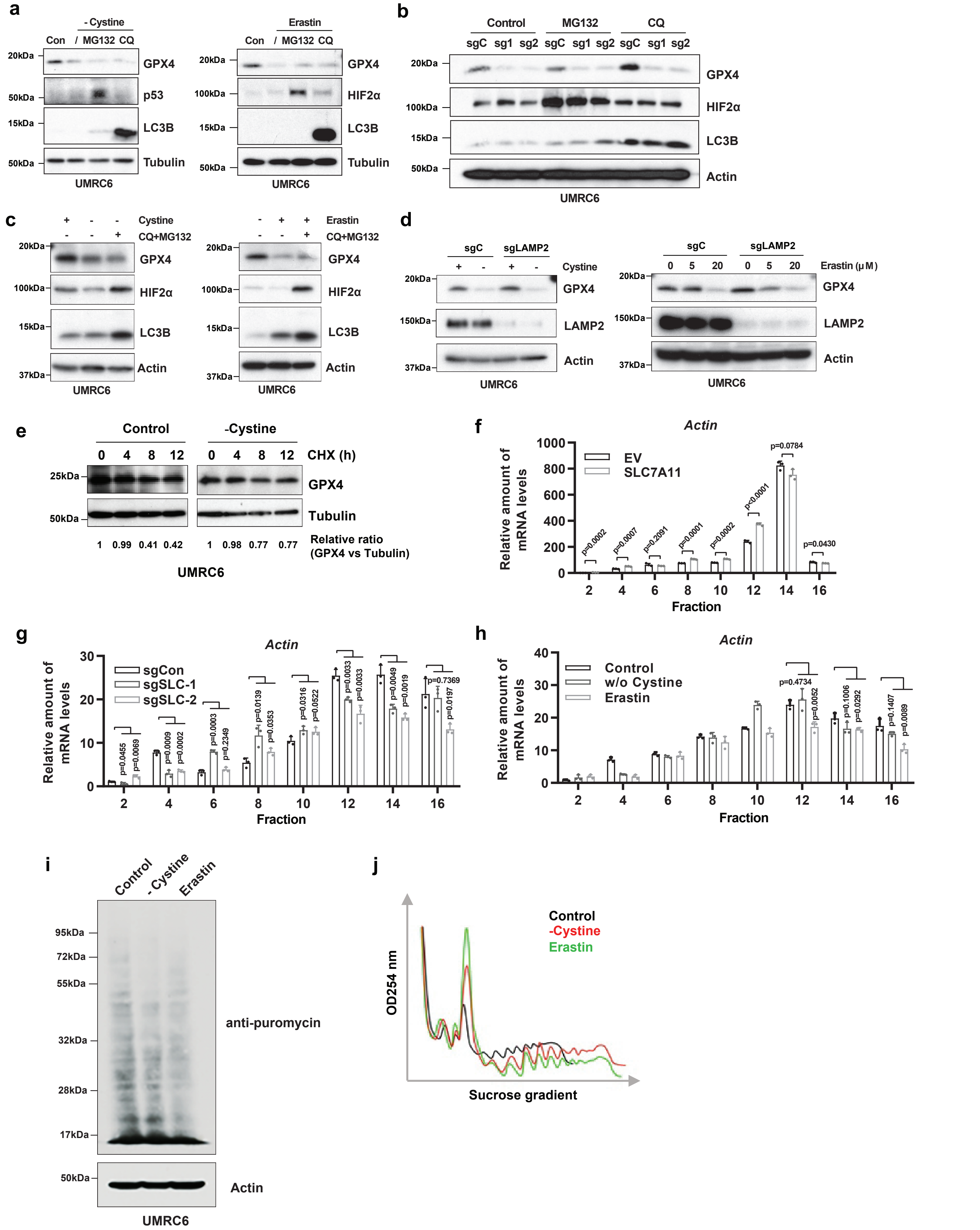

Supplementary Figure 2. SLC7A11-mediated cystine uptake regulates GPX4 independent of proteasome and/or autophagy-mediated protein degradation.

a URMC6 cells were treated in cystine-free or 10 μM erastin-contained media with or without 10 μM MG132 or 50 μM Chloroquine (CQ) for 24 hours followed by Western blotting analysis. **b** Western blotting analysis of control (sgC) and *SLC7A11*-KO (sg1 and sg2) UMRC6 cells cultured in media with or without 10 μM MG132 or 50 μM Chloroquine (CQ) for 16 hours. **c** URMC6 cells were cultured in cystine-free or 10 μM erastin-contained media with or without 5 μM MG132 and 25 μM Chloroquine (CQ) for 16 hours followed by Western blotting analysis. **d** UMRC6 control (sgC) and LAMP2-knockout (sgLAMP2) cells were cultured in cystine-free media or treated with indicated concentrations (μM) of erastin for 24 hours followed by Western blotting analysis. **e** UMRC6 cells cultured in media with or without cystine for 24 hours followed by adding 50 μg/ml cycloheximide (CHX) to each dish. Cells were collected at indicated time points and protein levels were analyzed by Western blotting. **f** 786-EV and -SLC cell lines were subjected to polyribosome fractionation followed by RT-PCR to analyze *ACTB* (Actin) mRNA distribution profiles during protein translation. n=3. **g** UMRC6-sgCon, -sgSLC-1 and -sgSLC-2 cells were subjected to polyribosome fractionation followed by RT-PCR to analyze *ACTB* (Actin) mRNA distribution profiles during protein translation. n=3. **h** UMRC6 cells were treated with control media, cystine-free media or erastin (10μM) for 24 hours followed by polyribosome fractionation and RT-PCR to analyze *ACTB* (Actin) mRNA distribution profiles during protein translation. n=3. **i** Protein synthesis assay for UMRC6 cell treated cystine-free media for 22 hours or 10 μM erastin for 24 hours. **j** Absorbance (A254 nm) of sucrose density gradient fractionated ribosomes from UMRC6 cells treated as described in **i**. For all panels, error bars are mean ± SD. n indicates biologically independent repeats. *P* value was determined by two-tailed unpaired Student's t-test. Source data are provided as a Source Data file.

Supplementary Figure 3

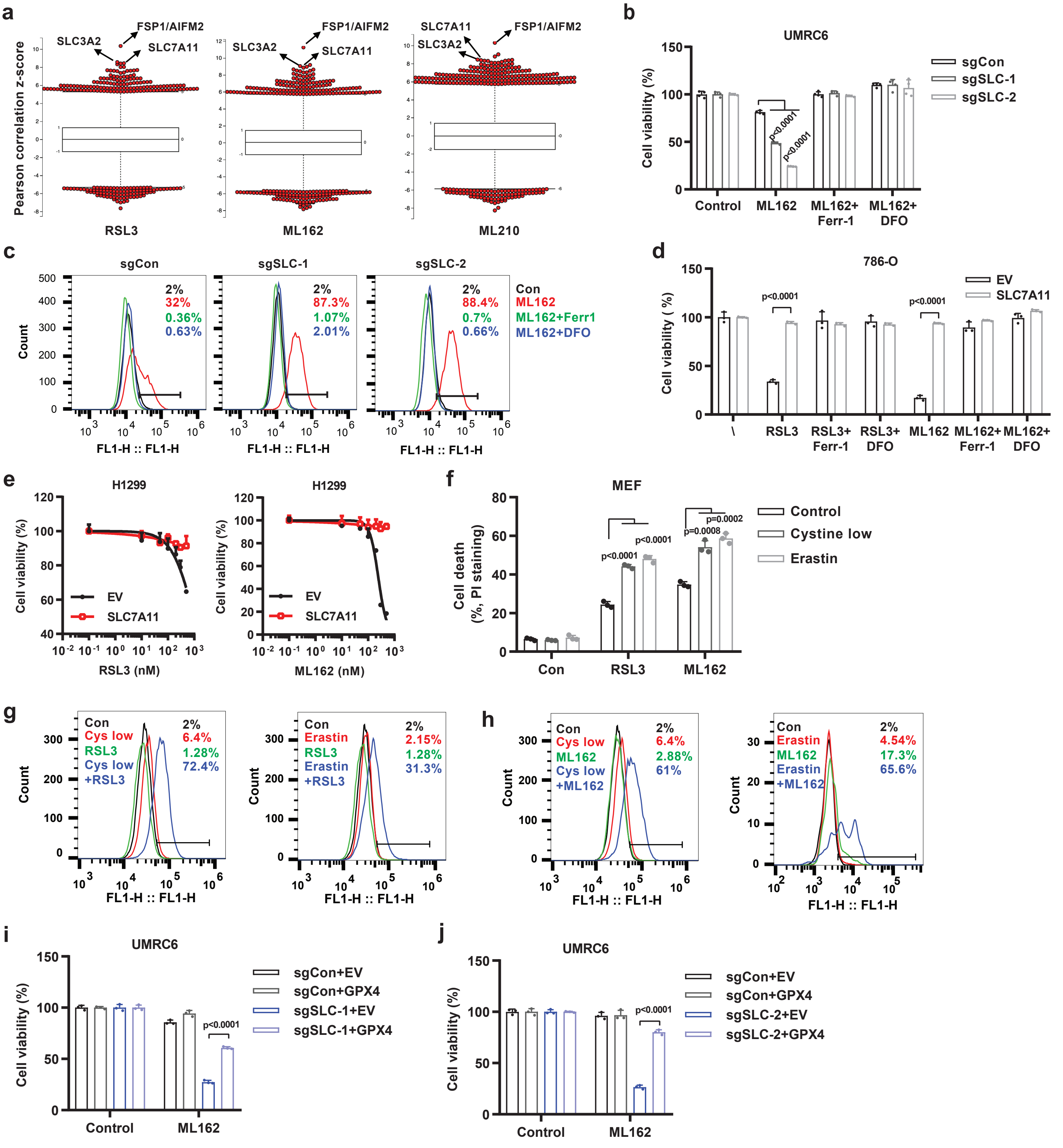

Supplementary Figure 3. SLC7A11 modulates ferroptosis sensitivity to class 2 FINs partly through regulating GPX4 levels.

a Correlation between the sensitivity to indicated drugs and gene expression in 860 cancer cell lines (https://portals.broadinsitute.org/ctrp). **b** Cell viability was determined for control (sgCon) and SLC7A11-knockout (sgSLC-1 and sgSLC-2) UMRC6 cells treated with 500 nM ML162 combined with or without 5 μM Ferrostatin-1 (Ferr-1) or 100 μM deferoxamine (DFO) for 10 hours. n=4. **c** UMRC6 cells were treated with 600 nM ML162 combined with or without 5 μM Ferrostatin-1 (Ferr-1) or 100 μM deferoxamine (DFO) for 6 hours. Then lipid peroxidation was assessed using BODIPY™ 581/591 C11 staining followed by FACS analysis. **d** 786-O-EV and -SLC7A11 cells treated with 400nM RSL3 or ML162 with or without 5 μM Ferrostatin-1 (Ferr-1) or 100 μM deferoxamine (DFO) for 9 hours followed by cell viability analysis. n=3. **e** Empty vector- (EV) and SLC7A11-overexpressing (SLC7A11) H1299 cells were treated with RSL3 or ML162 at indicated concentrations for 9 hours followed by cell viability analysis. n=3. **f** MEFs were cultured in control, cystine low (1 μM) or erastin (0.5 μM) with or without RSL3 (400 nM) or ML162 (400 nM) for 10 hours followed by PI staining and FACS analysis. n=3. **g** Left, UMRC6 cells were cultured in cystine-low (1 μM) media combined with or without 300 nM RSL3 for 6 hours. Right, UMRC6 cells were treated with Erastin (5 μM) combined with or without 300 nM RSL3 for 6 hours. Then lipid peroxidation was assessed using BODIPY™ 581/591 C11 staining followed by FACS analysis. **h** Left, UMRC6 cells were cultured in cystine-low (1 μM) media combined with or without 300 nM ML162 for 6 hours. Right, UMRC6 cells were treated with Erastin (5 μM) combined with or without 300 nM ML162 for 6 hours. Then lipid peroxidation was assessed using BODIPY™ 581/591 C11 staining followed by FACS analysis. **i-j** Cell viability of indicated cell treated with 400 nM ML162 for 8 hours. n=3. For all panels, error bars are mean ± SD. n indicates biologically independent repeats. *P* value was determined by two-tailed unpaired Student's t-test. Source data are provided as a Source Data file.

Supplementary Figure 4

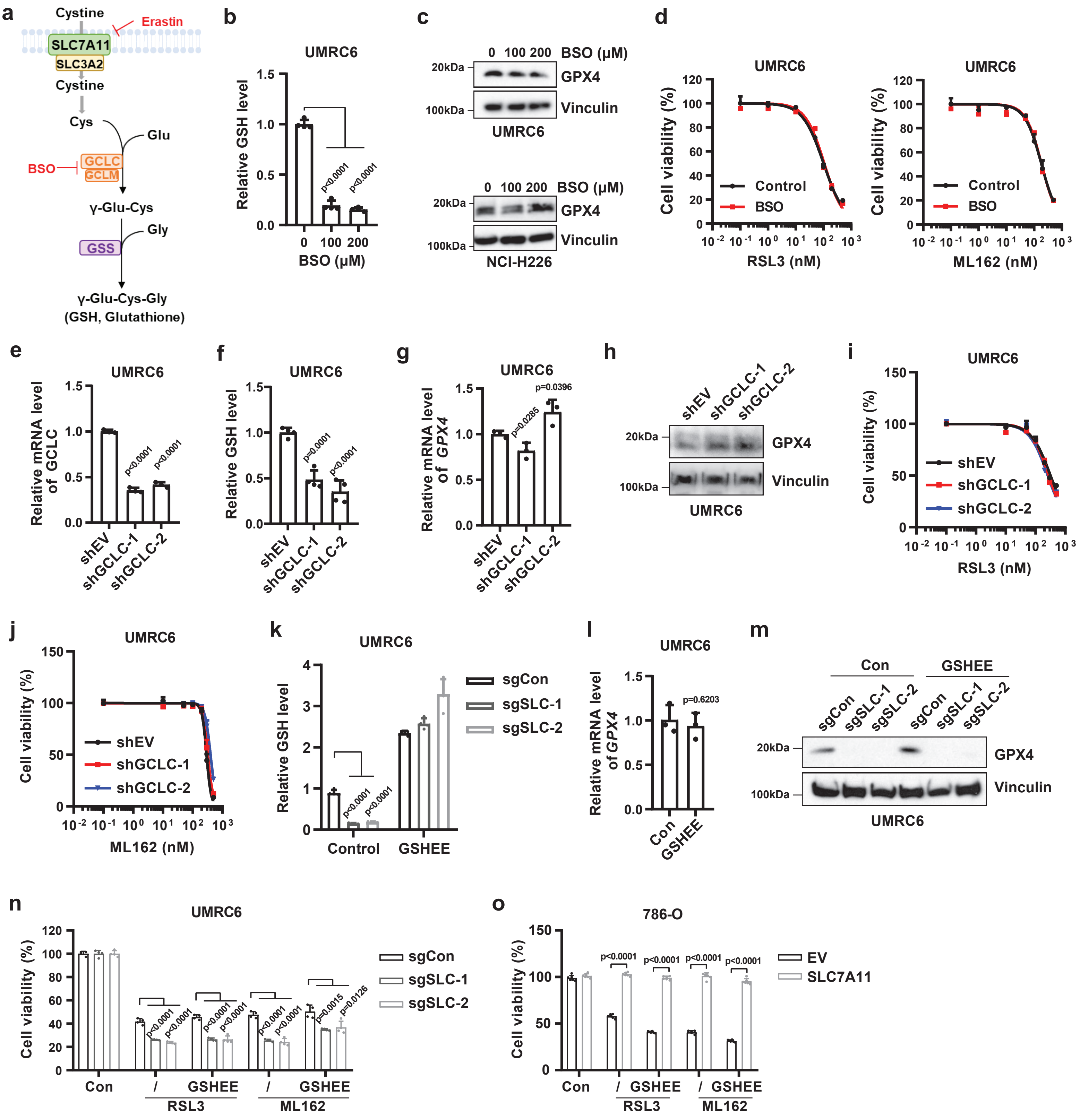

Supplementary Figure 4. GSH depletion does not regulate GPX4 protein levels or ferroptosis sensitivity to class 2 FINs.

a Diagram showing glutathione synthesis pathway. BSO, buthionine sulfoximine. **b** Bar graph showing intracellular GSH levels in UMRC6 cells treated with BSO at indicated concentrations for 24 hours. n=4. **c** Western blotting analysis of indicated cells treated with BSO at indicated concentrations for 24 hours. **d** Cell viability measured in UMRC6 cells pretreated with 100 μM BSO for 24 hours followed by treatment with different concentration of RSL3 or ML162 for 10 hours. **e** RT-PCR analysis of *GCLC* mRNA levels in the indicated cell lines. n=3. **f** Bar graph showing intracellular GSH levels in the indicated cells. n=4. **g** RT-PCR analysis of *GPX4* mRNA levels in the indicated cell lines. n=3. **h** GPX4 protein expression level analyzed by Western blotting in indicated cells. **i** Cell viability of indicated cells treated with RSL3 at different concentrations for 8 hours. **j** Cell viability of indicated cells treated with ML162 at different concentrations for 10 hours. **k** Bar graph showing intracellular GSH levels in indicated cells treated with 5 μM Glutathione ethylester (GSHEE) for 24 hours. n=4. **l** RT-PCR analysis of *GPX4* mRNA levels in UMRC6 cells treated with or without 5 μM Glutathione ethylester (GSHEE) for 24 hours. n=3. **m** GPX4 protein expression levels analyzed by Western blotting in indicated cells treated with or without 5 μM Glutathione ethylester (GSHEE) for 24 hours. **n** Cell viability of indicated cells treated with 1 μM RSL3 or ML162 combined with or without 5 μM GSHEE for 8 hours. n=4. **o** Cell viability of indicated cells treated with 0.5 μM RSL3 or ML162 combined with or without 5 μM GSHEE for 8 hours. n=6. For all panels, error bars are mean ± SD. n indicates biologically independent repeats. *P* value was determined by two-tailed unpaired Student's t-test. Source data are provided as a Source Data file.

Supplementary Figure 5

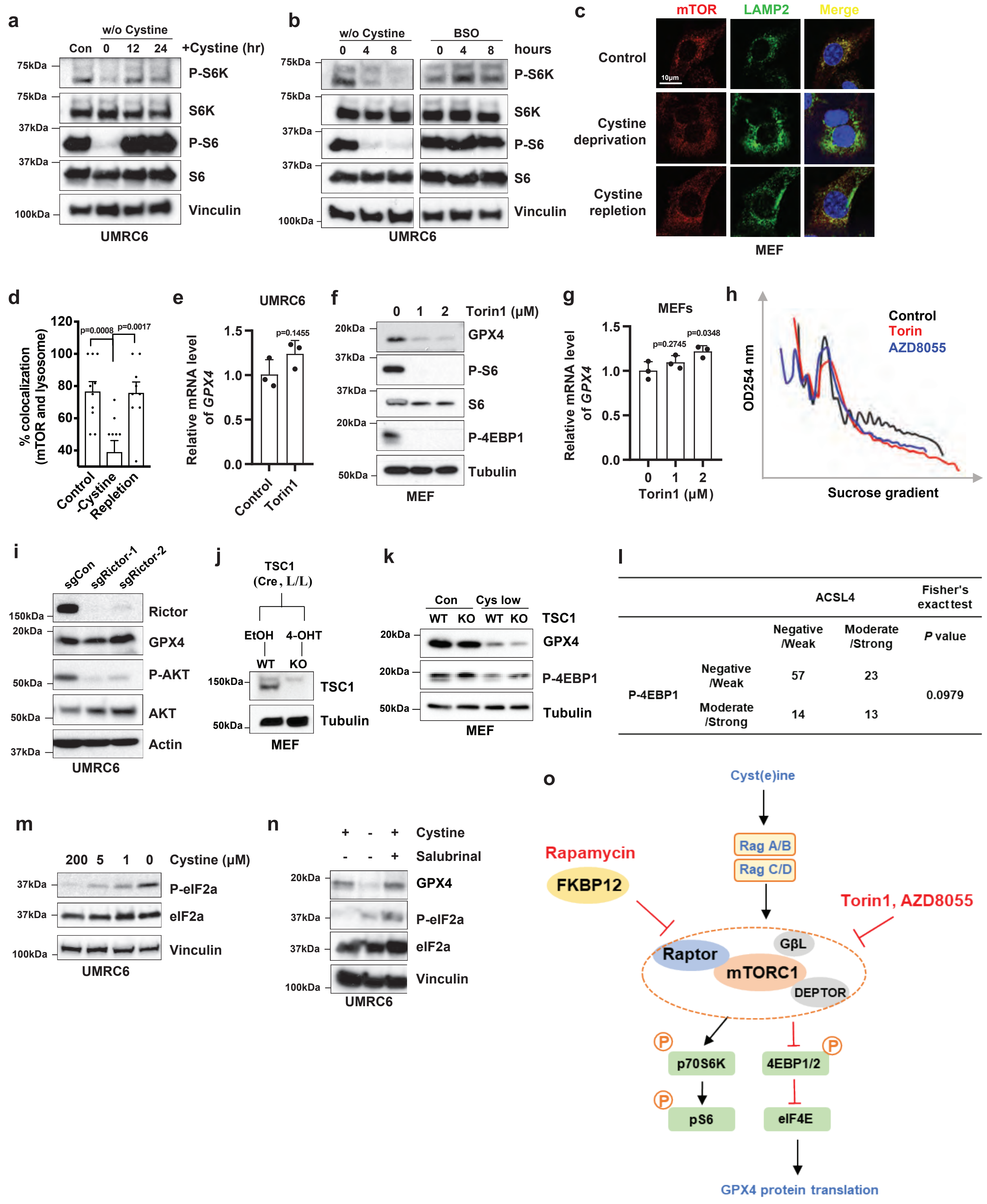

Supplementary Figure 5. Cyst(e)ine promotes GPX4 protein synthesis partly through mTORC1-4E-BP signaling.

a UMRC6 cells were cultured in cystine-free media for 24 hours. Then 200 μM cystine was added back to the media for indicated time followed by Western blotting. **b** UMRC6 cells were cultured in cystine-free media or 100 μM BSO-contained media for indicated time followed by Western blotting. **c** MEFs were cultured in cystine-free media for 8 hours (Cystine deprivation). Then 200 μM cystine was added back to the media for 12 hours (Cystine repletion) followed by immunofluorescence imaging. **d** Quantification of percentage for cells with colocalized mTOR and LAMP2 in MEFs treated as described in **c**. n=9 randomly observed areas per slides of treated sample. Error bars are mean ± SE. *P* value was determined by two-tailed unpaired Student's t-test. **e** RT-PCR analysis of *GPX4* mRNA level in UMRC6 cells treated with or without 200 nM Torin1 for 24 hours. n=3. **f** MEFs were treated with Torin1 at indicated concentrations for 24 hours followed by Western blotting analysis. **g** RT-PCR analysis for *GPX4* mRNA levels in MEFs treated with Torin1 at indicated concentrations for 24 hours. n=3. **h** Absorbance (A254 nm) of sucrose density gradient fractionated ribosomes from control, Torin1 (1 μM)- or AZD8055 (1 μM)-treated cell extracts. **i** Protein levels in UMRC6 control (sgC) and Rictor knockout cells (sgRictor-1 and sgRictor-2) were analyzed by Western blotting. **j** TSC1-knockout MEFs were generated as shown in the workflow and verified using Western blotting. **k** Cells were cultured in control or cystine low (5 μM cystine) media for 16 hours followed by Western blotting analysis. **l** The expression analysis of ACSL4 and p-4EBP1 in tissue microarrays. **m** UMRC6 cells were cultured in media with indicated concentrations of cystine for 24 hours followed by Western blotting analysis. **n** UMRC6 cell were cultured in cystine-free media or treated with 10 μM salubrinal for 24 hours followed by Western blotting analysis. **o** Diagram of mTORC1/4EBPs pathway regulation on protein translation. For panel e and g, error bars are mean ± SD. n indicates biologically independent repeats. *P* value was determined by two-tailed unpaired Student's t-test. Source data are provided as a Source Data file.

Supplementary Figure 6

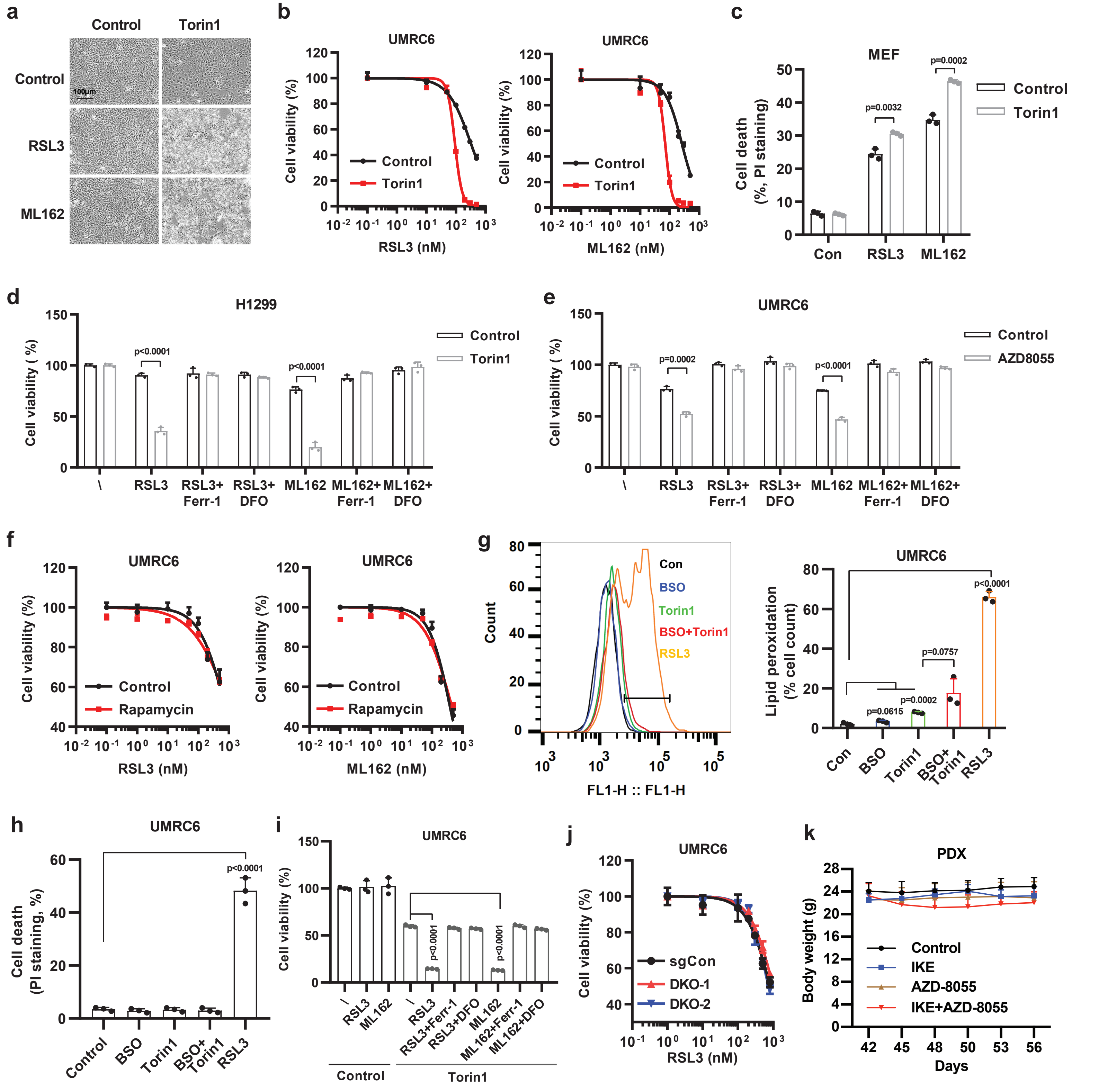

Supplementary Figure 6. mTORC1 inhibition sensitizes cancer cells or tumors to ferroptosis. **a** Representative phase-contrast images of UMRC6 cells treated with 400 nM RSL3 or ML162 in the presence of 1 μM Torin1 or not for 7 hours. **b** Cell viability of UMRC6 cells treated with RSL3 or ML162 at indicated concentrations combined with 200 nM Torin1 or not for 10 hours. **c** MEFs were cultured in media with 500 nM RSL3 or ML162 in the presence of 1 μM Torin1 or not for 16 hours. Then cell death was determined by PI staining followed by FACS analysis. n=3. **d** H1299 cells were treated with 300 nM RSL3 or ML162 combined with 1 μM Torin1, and/or 5 μM Ferr-1 or 100 μM DFO for 8 hours followed by cell viability analysis. n=3. **e** UMRC6 cells were treated with 400 nM RSL3 or ML162 combined with 1 μM AZD8055, and/or 5 μM Ferr-1 or 100 μM DFO for 8 hours followed by cell viability analysis. n=3. **f** Cell viability of UMRC6 cells treated with RSL3 or ML162 at indicated concentrations combined with 200 nM rapamycin or not for 10 hours. **g** UMRC6 cells were treated with BSO (100 μM), Torin1 (1 μM) or BSO and Torin1 together for 24 hours or RSL3 (0.5 μM) for 6 hours followed by lipid peroxidation analysis using Bodipy 581/591 C11. Bar graph showing quantitative analysis of lipid peroxidation levels. n=3. **h** UMRC6 cells were treated as described in **g** followed by PI staining for cell death analysis. n=3. **i** UMRC6 cells were pretreated with 1 μM Torin1 for 24 hours. Then cells were treated wtih indicated combinations for 8 hours followed by cell viability analysis. RSL3 and ML162, 400 nM; Ferr-1, 5 μM; DFO, 100 μM. n=3. **j** Cell viability of control (sgCon) and 4EBP1/2 double-knockout (DKO-1 and DKO-2) UMRC6 cells treated with RSL3 at indicated concentrations for 10 hours. **k** Body weights of PDX tumor-bearded mice injected daily with 30 mg/kg IKE, or 10mg/kg AZD8055, or both at different time points as shown. For all panels, error bars are mean ± SD. n indicates biologically independent repeats. *P* value was determined by two-tailed unpaired Student's t-test. Source data are provided as a Source Data file.

Supplementary Figure 7

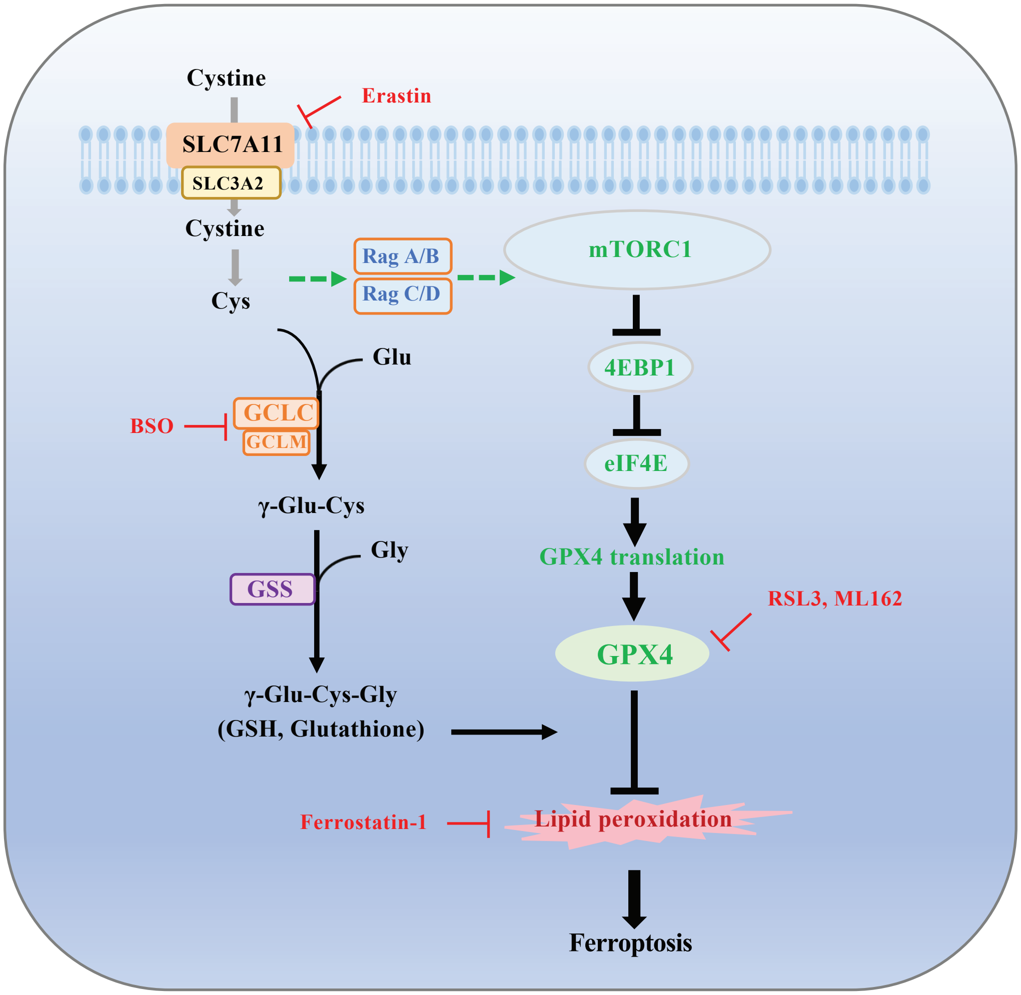

Supplementary Figure 7. The model depicting translational regulation of GPX4 by cyst(e)ine and mTORC1-4E-BP signaling.

Supplementary Figure 8

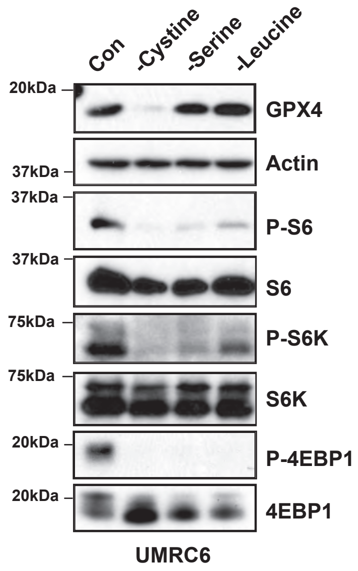

Supplementary Figure 8. Western blotting showing GPX4 level and mTOR1 signaling upon starvation of different amino acids as indicated for 24 hours in UMRC6 cells.

Supplementary Figure 9

Gating example for lipid peroxidation analysis

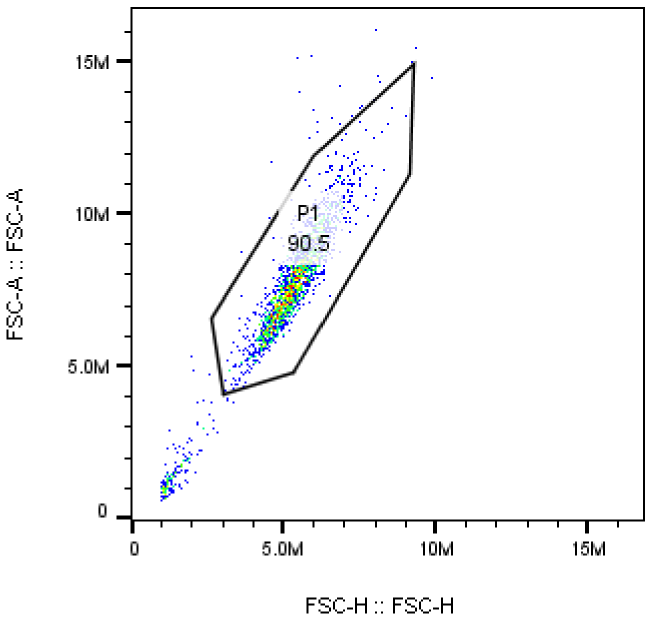

Gating example for PI staining-mediated cell death analysis

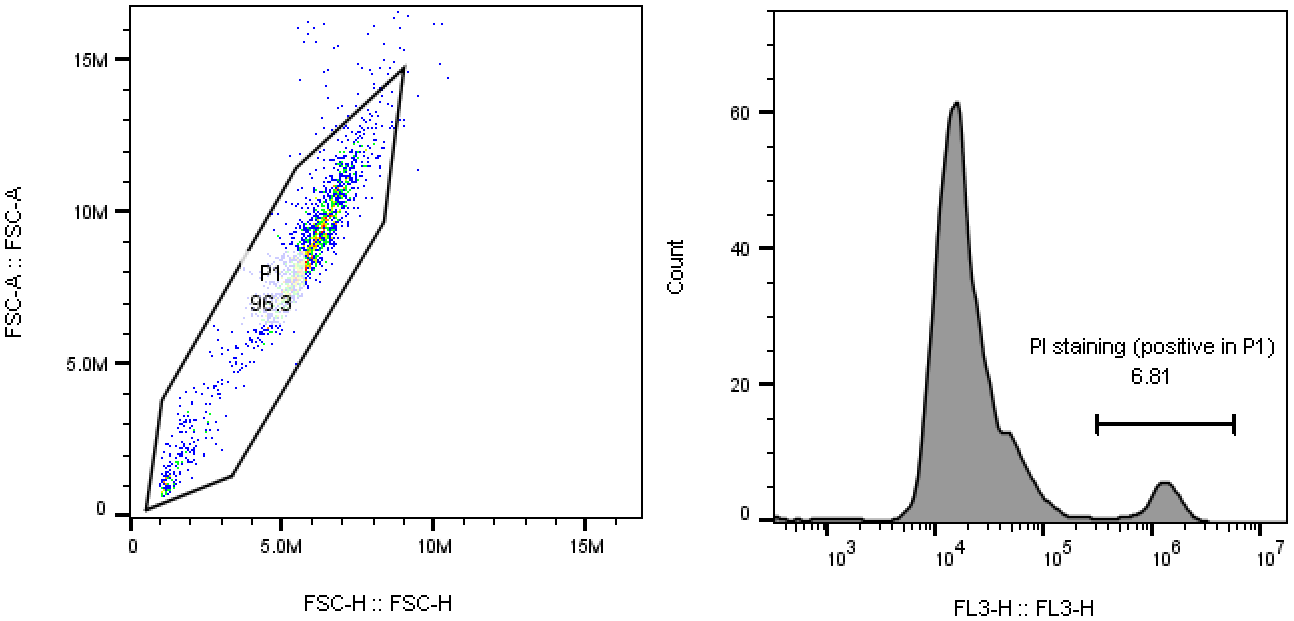

Supplementary Figure 9. Gating strategies for lipid peroxidation and PI staining analysis through flow cytometry.

Supplementary Figure 10 Uncropped blots

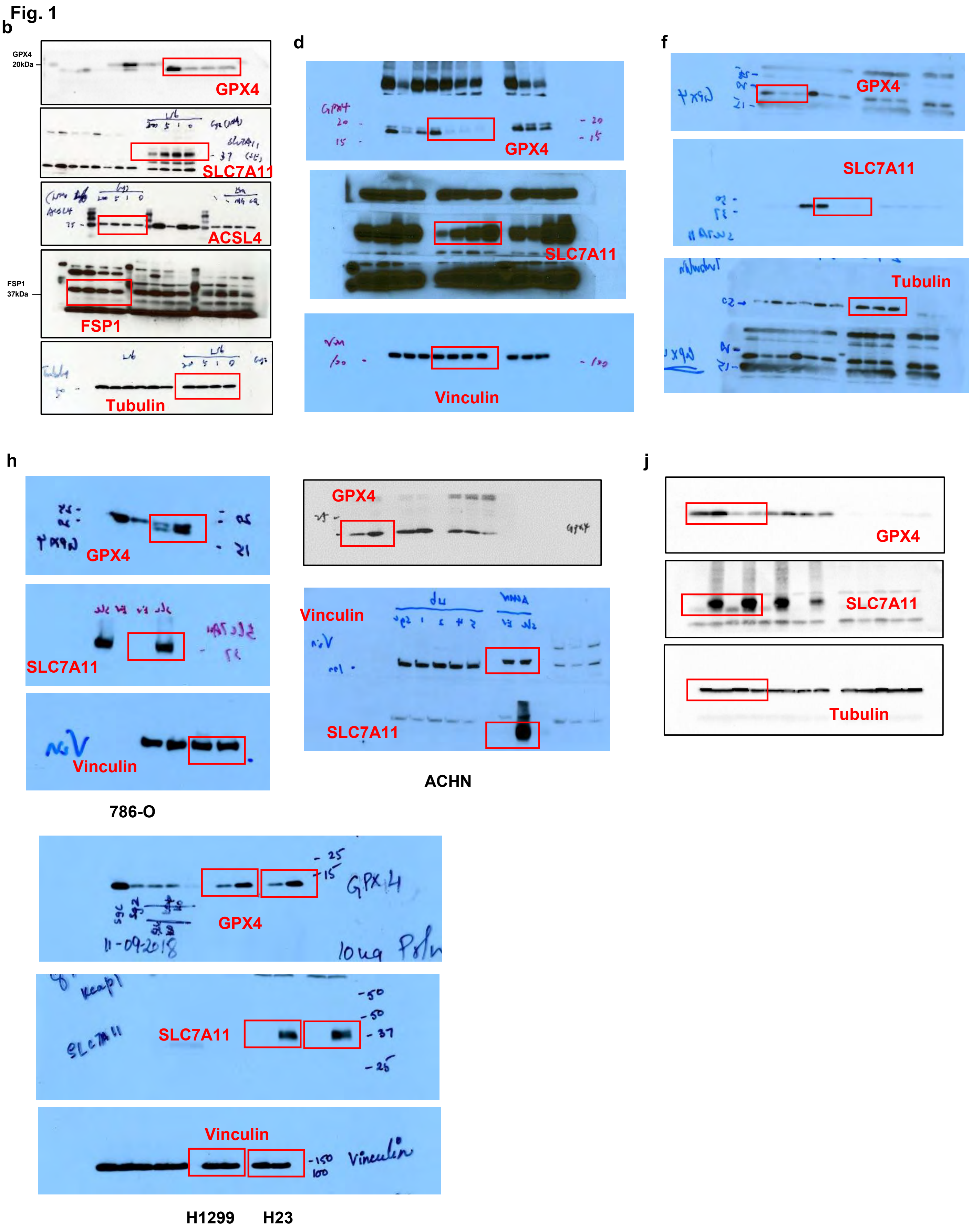

Supplementary Figure 10 Uncropped blots

Fig. 2

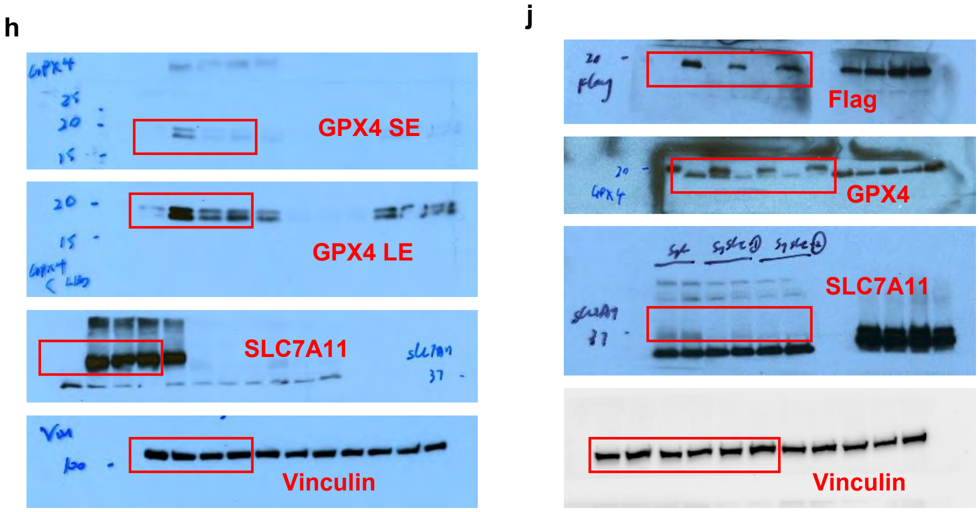

Supplementary Figure 10 Uncropped blots

Fig. 3

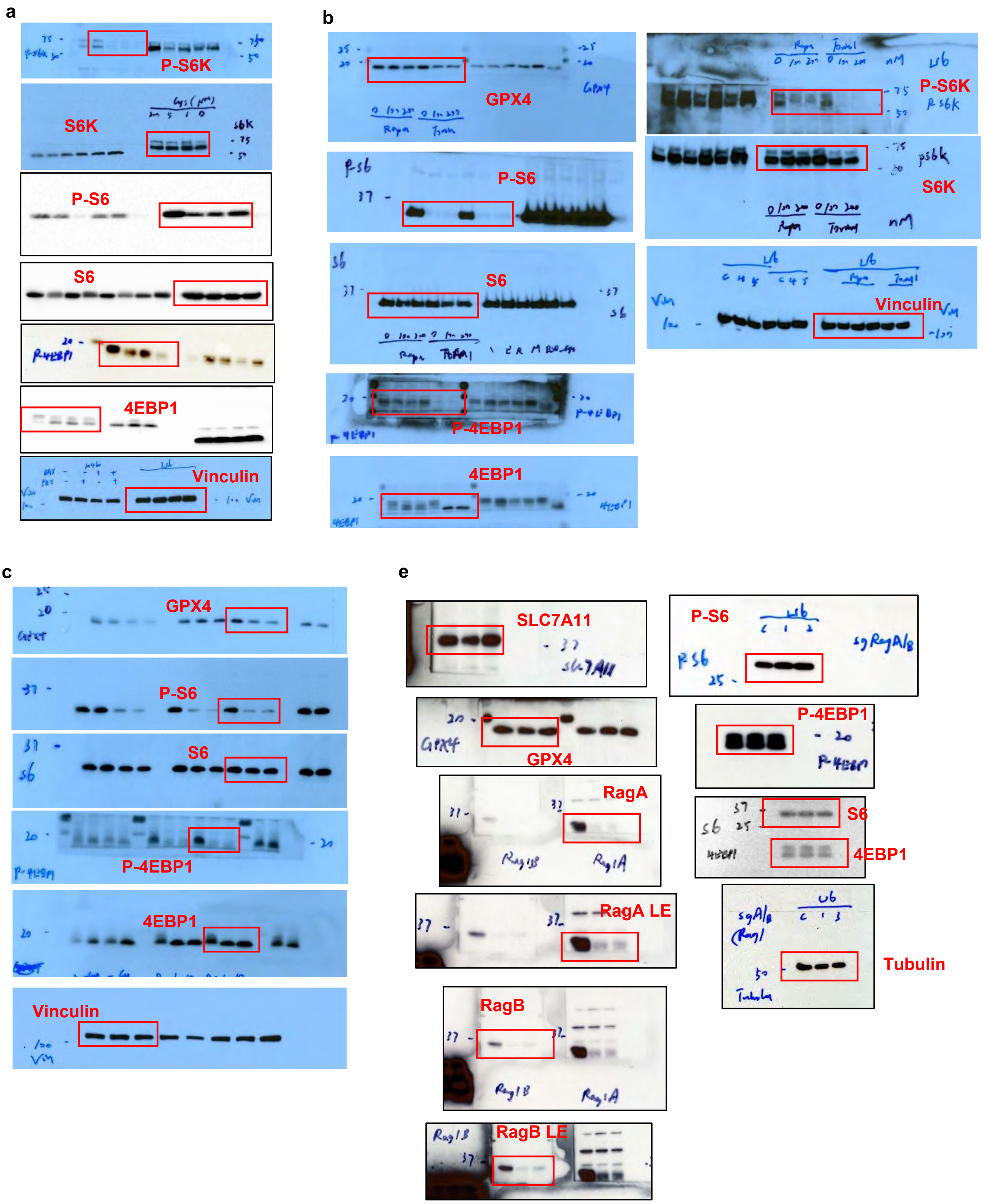

Supplementary Figure 10 Uncropped blots

Fig. 3 continued

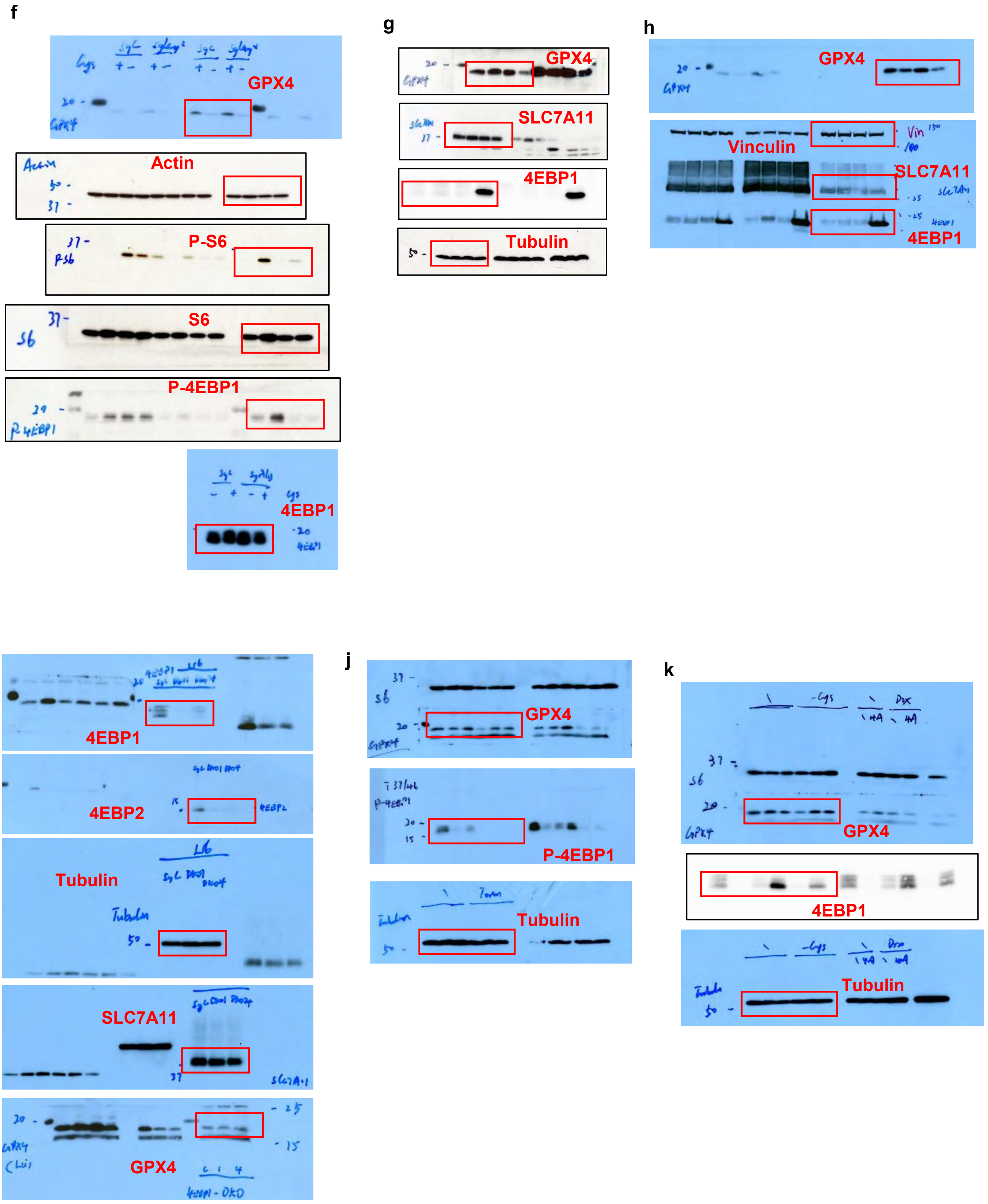

Supplementary Figure 10 Uncropped blots

Fig. S1

c

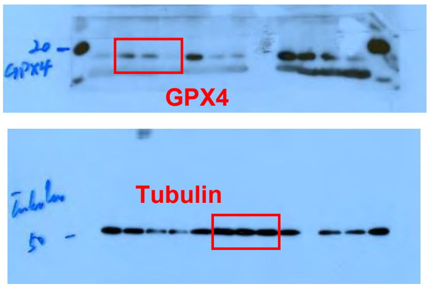

e

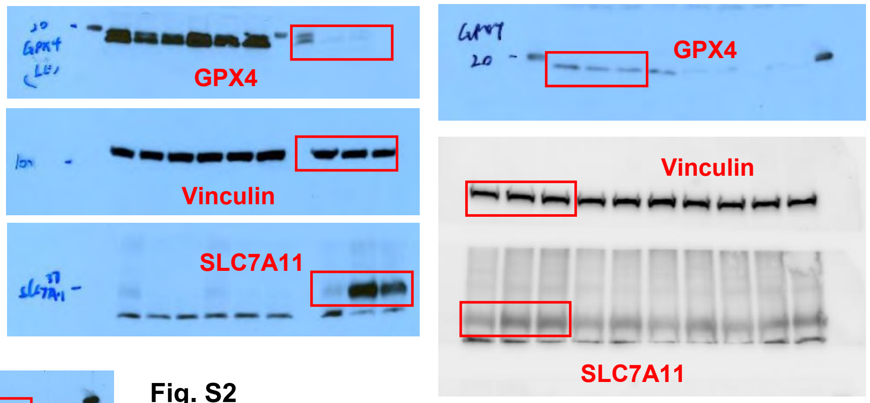

i

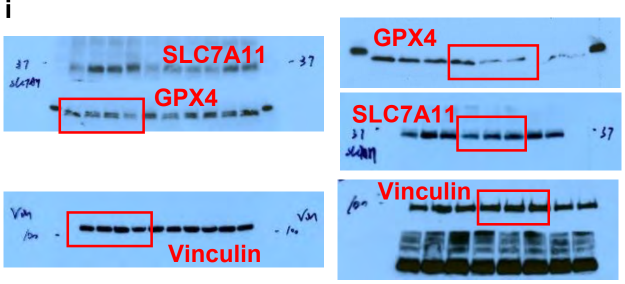

H226 H460

m

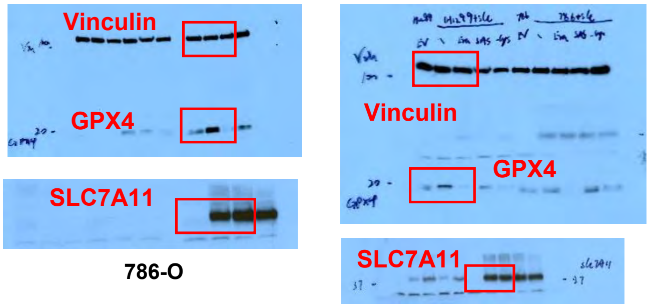

786-O H1299

Fig. S2

a

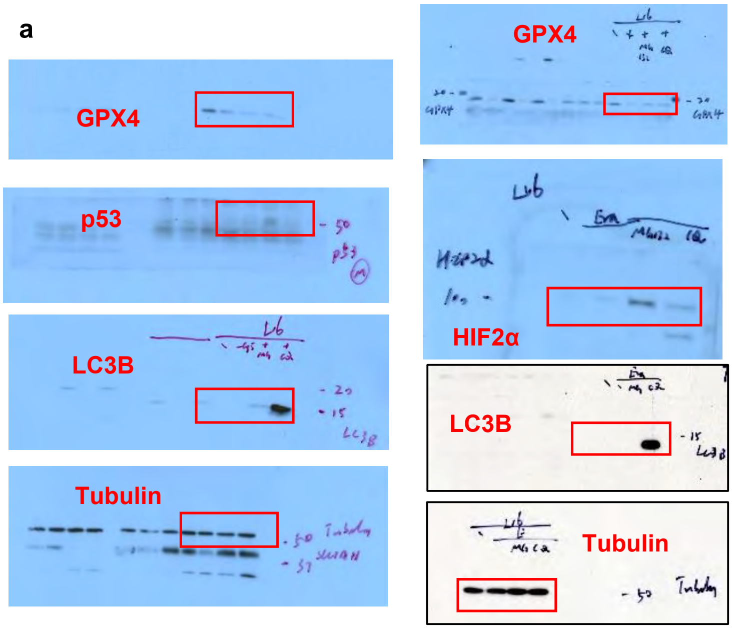

Fig. S2

b

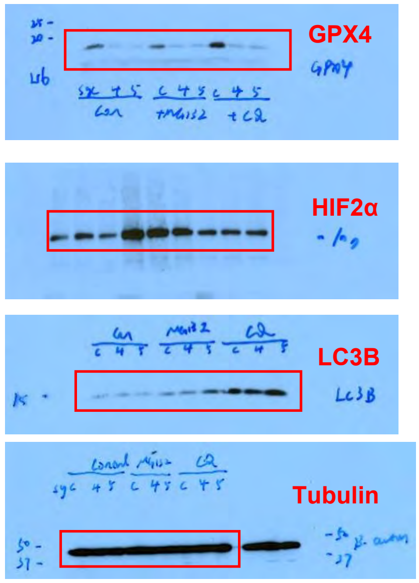

c

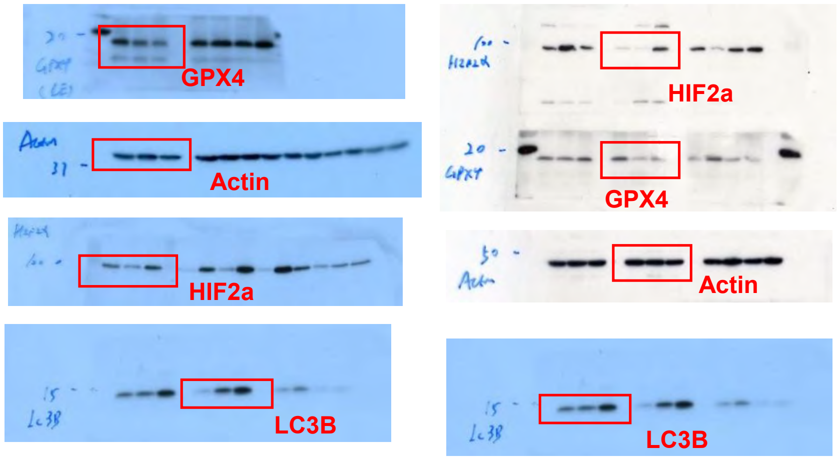

Supplementary Figure 10 Uncropped blots

Fig. S2 **d**

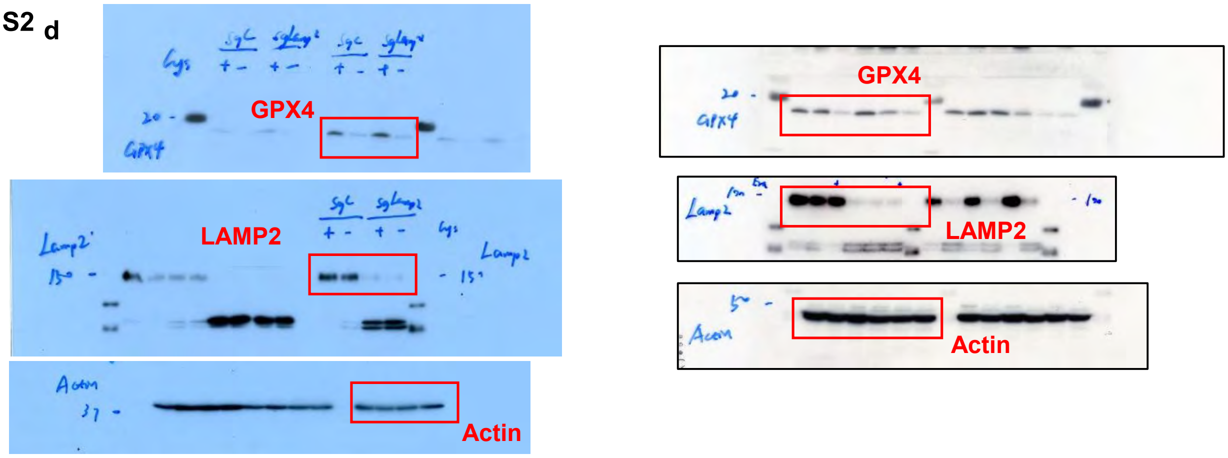

e

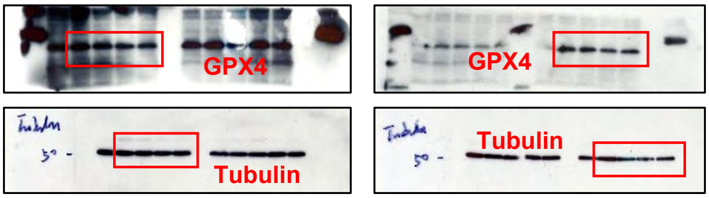

i

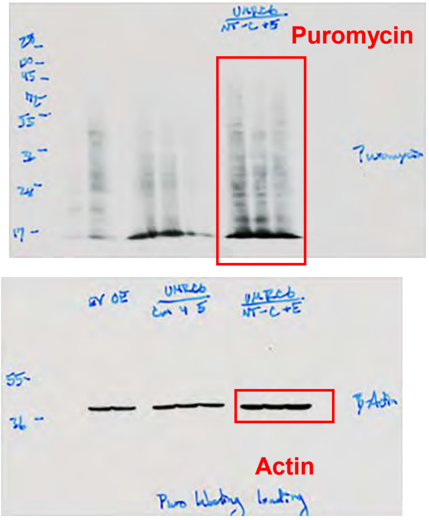

Fig. S4

c

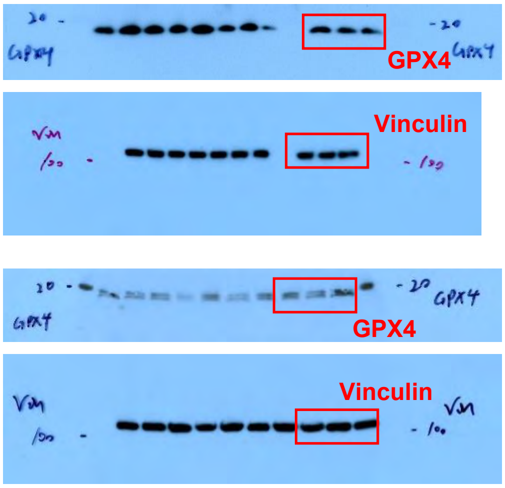

h

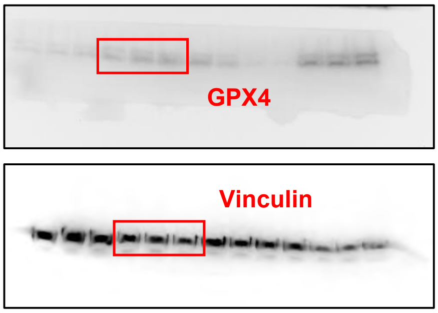

m

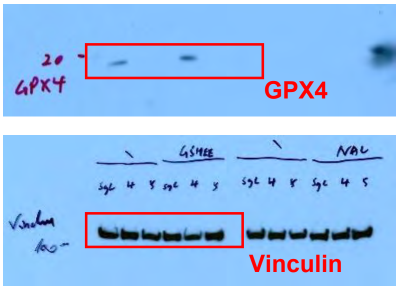

Supplementary Figure 10 Uncropped blots

Fig. S5

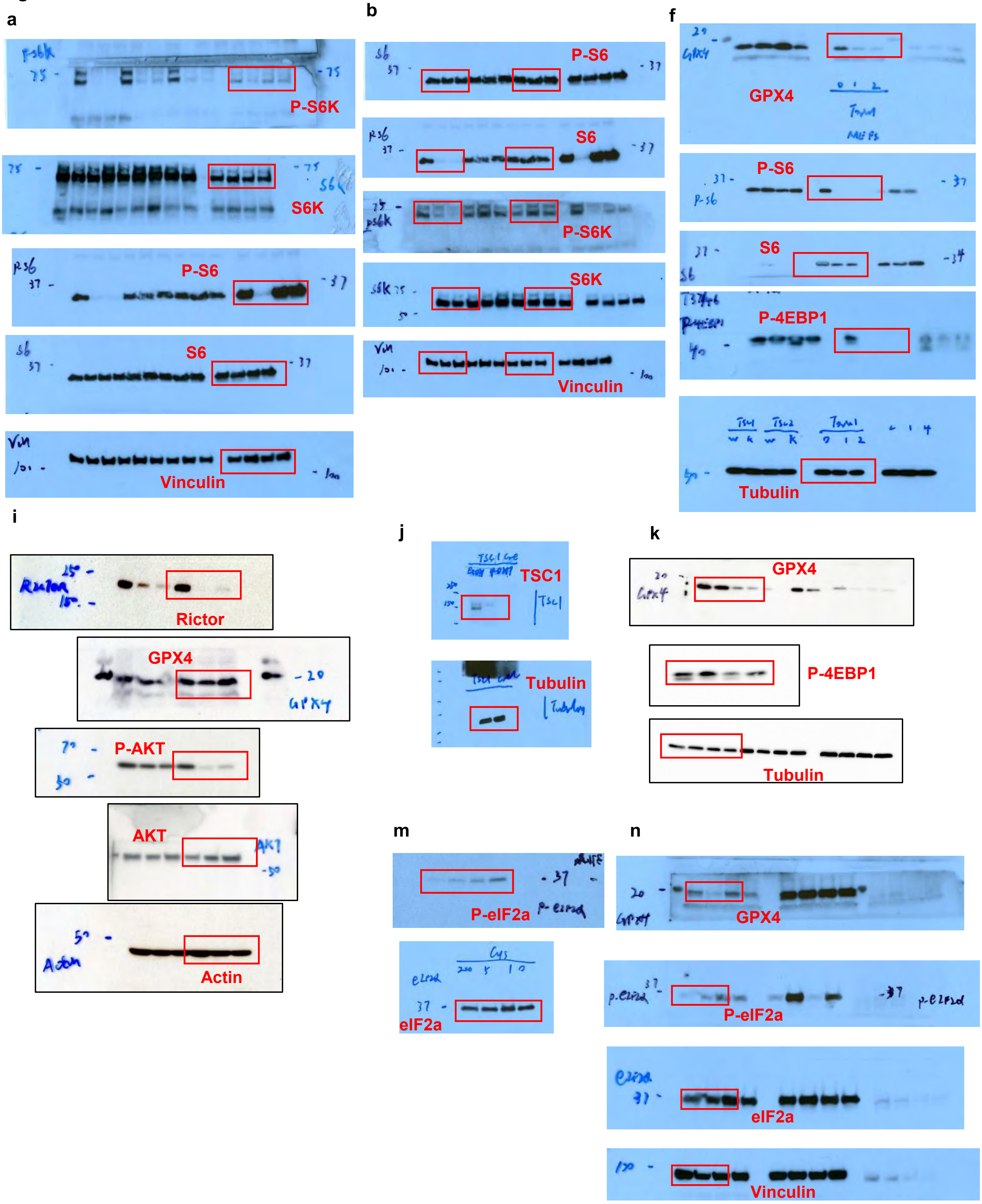

Supplementary Figure 10 Uncropped blots

Fig. S8

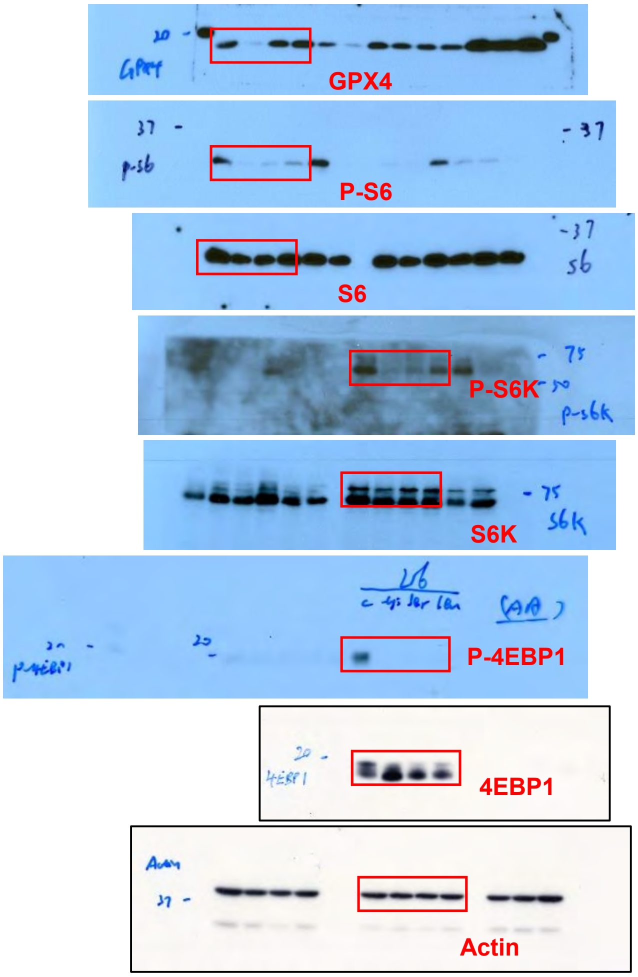

Supplement: Supplementary file 1 — Supplementary information [file 41467_2021_21841_MOESM1_ESM.pdf]
