# Supplementary material for: mTORC1 couples cyst(e)ine availability with GPX4 protein synthesis and ferroptosis regulation
Source: Nat Commun. 2021 Mar 11;12:1589. doi: 10.1038/s41467-021-21841-w (PMC7952727; doi:10.1038/s41467-021-21841-w)
Supplement: Supplementary file 6 — Description of additional supplementary files [file 41467_2021_21841_MOESM6_ESM.docx]

Description of additional supplementary information

Title: Supplementary Table 1.

Description: List of significantly down- and up-regulated proteins in UMRC6 cells cultured in 1 μM cystine media compared with control.

Title: Supplementary Table 2.

Description: Whole protein list in UMRC6 cells cultured in control or 1 μM cystine media identified through mass spectrometry.

Title: Supplementary Table 3.

Description: Sequences of oligos used in this study.
