# Supplementary material for: mTORC1 couples cyst(e)ine availability with GPX4 protein synthesis and ferroptosis regulation
Source: Nat Commun. 2021 Mar 11;12:1589. doi: 10.1038/s41467-021-21841-w (PMC7952727; doi:10.1038/s41467-021-21841-w)
Supplement: Supplementary file 7 — Reporting Summary [file 41467_2021_21841_MOESM7_ESM.pdf]

nature research

Corresponding author(s): Boyi Gan

Last updated by author(s): 01/29/2021

Reporting Summary

Nature Research wishes to improve the reproducibility of the work that we publish. This form provides structure for consistency and transparency in reporting. For further information on Nature Research policies, see our Editorial Policies and the Editorial Policy Checklist.

Statistics

For all statistical analyses, confirm that the following items are present in the figure legend, table legend, main text, or Methods section.

n/a	Confirmed	
☐	☒	The exact sample size (*n*) for each experimental group/condition, given as a discrete number and unit of measurement
☐	☒	A statement on whether measurements were taken from distinct samples or whether the same sample was measured repeatedly
☐	☒	The statistical test(s) used AND whether they are one- or two-sided<br>*Only common tests should be described solely by name; describe more complex techniques in the Methods section.*
☒	☐	A description of all covariates tested
☐	☒	A description of any assumptions or corrections, such as tests of normality and adjustment for multiple comparisons
☒	☐	A full description of the statistical parameters including central tendency (e.g. means) or other basic estimates (e.g. regression coefficient) AND variation (e.g. standard deviation) or associated estimates of uncertainty (e.g. confidence intervals)
☐	☒	For null hypothesis testing, the test statistic (e.g. *F*, *t*, *r*) with confidence intervals, effect sizes, degrees of freedom and *P* value noted<br>*Give P values as exact values whenever suitable.*
☒	☐	For Bayesian analysis, information on the choice of priors and Markov chain Monte Carlo settings
☒	☐	For hierarchical and complex designs, identification of the appropriate level for tests and full reporting of outcomes
☒	☐	Estimates of effect sizes (e.g. Cohen's *d*, Pearson's *r*), indicating how they were calculated

Our web collection on statistics for biologists contains articles on many of the points above.

Software and code

Policy information about availability of computer code

Data collection	Accuri C6 (BD Bioscience) was used to collect flow cytometry data.
Data analysis	FlowJo_Vl0 was used for flow cytometry data analysis. MaxQuant software (version 1.6.5.0) was used for analyzing data from Q Exactive HF-X mass spectrometer. Peptides information was searched against the human proteomes database from public database Uniprot. The data of protein information was analyzed by Perseus (version 1.6.7.0) and significantly regulated targets were identified. All the plots for in vitro cell line treatment were generated by GraphPad Prism (Version 8.4.3).

For manuscripts utilizing custom algorithms or software that are central to the research but not yet described in published literature, software must be made available to editors and reviewers. We strongly encourage code deposition in a community repository (e.g. GitHub). See the Nature Research guidelines for submitting code & software for further information.

Data

Policy information about availability of data

All manuscripts must include a data availability statement. This statement should provide the following information, where applicable:

- Accession codes, unique identifiers, or web links for publicly available datasets
- A list of figures that have associated raw data
- A description of any restrictions on data availability

The raw data and processed data for mass spectrometry analysis of proteins in UMRC6 cells upon cystine deprivation have been deposited to MassIVE datasets (ftp://massive.ucsd.edu/MSV000086009/). Uniprot is a public and freely accessible resource of protein sequence and functional information (https://www.uniprot.org/). The uncropped films for immunoblots used in this study have been shown in supplementary figures. The raw data used for generating graphs are included in Source Data file. All other data that support the findings of this study are available from the corresponding author upon reasonable request.

Field-specific reporting

Please select the one below that is the best fit for your research. If you are not sure, read the appropriate sections before making your selection.

☒ Life sciences ☐ Behavioural & social sciences ☐ Ecological, evolutionary & environmental sciences

For a reference copy of the document with all sections, see nature.com/documents/nr-reporting-summary-flat.pdf

Life sciences study design

All studies must disclose on these points even when the disclosure is negative.

Sample size	No sample size calculations were performed. Sample size was determined according to our experience as described in our previous publications (Y Zhang, 2018, PMID: 30202049).
Data exclusions	No samples or animals were excluded from the analysis.
Replication	At least 3 independent repeats were included for related experiments. Each experiment was performed for at least twice to make sure similar results are reproducible.
Randomization	6-8 week female mice were used for xenograft hosts. After inoculated with patient-derived tumor tissues, tumor growth was monitored over weeks. Then mice with similar size of tumor burden were randomly allocated into experimental groups for further treatment.
Blinding	For cell-based experiments, western blotting, FACS and animal experiments, cell types or treatments were known when prepare the samples or start treatment at the beginning of experiments. Mass spectrometry analysis were blinded before analysis.

Reporting for specific materials, systems and methods

We require information from authors about some types of materials, experimental systems and methods used in many studies. Here, indicate whether each material, system or method listed is relevant to your study. If you are not sure if a list item applies to your research, read the appropriate section before selecting a response.

Materials & experimental systems

n/a	Involved in the study	
☐	☒	Antibodies
☐	☒	Eukaryotic cell lines
☒	☐	Palaeontology and archaeology
☐	☒	Animals and other organisms
☐	☒	Human research participants
☒	☐	Clinical data
☒	☐	Dual use research of concern

Methods

n/a	Involved in the study	
☒	☐	ChIP-seq
☐	☒	Flow cytometry
☒	☐	MRI-based neuroimaging

Antibodies

Antibodies used	GXP4 (1:1000, R&D systems, MAB5457), tubulin (1:5000; Cell Signaling Technology, #2144), vinculin (1:10000; Sigma, V4505), SLC7All (1:4000; Cell Signaling Technology, #12691) p53 (1:1000; Santa Cruze, sc-126), LC3B (1:5000; Cell Signaling Technology, #3868), HIF2a (1:1000; Cell Signaling Technology, #7096), TSCl (1:1000; Cell Signaling Technology, #6935) phospho-S6K (1:1000; Cell Signaling Technology, #9205), S6K (1:1000; Cell Signaling Technology, #9202), P-S6 (1:1000; Cell Signaling Technology, #2215), S6 (1:1000; Cell Signaling Technology, #2217), P-4EBP1 (1:1000; Cell Signaling Technology, #2855), 4EBP1 (1:1000; Cell Signaling Technology, #9644), 4EBP2 (1:1000; Cell Signaling Technology, #2845), Actin (1:1000; Cell Signaling Technology, #3700), Rictor (1:1000; Cell Signaling Technology, #2140), puromycin (Developmental Studies Hybridoma Bank, 1:1000, #PMY-2A4), β-Actin (Sigma-Aldrich, 1:5000, A2228)
Validation	All antibodies used in our study have been validated and detailed information could be found on the website from manufactures as listed below. Some of them have also been validated by our experiments as shown in this manuscript using either overexpress, knockout or knockdown strategies.<br>GXP4 (1:1000, R&D systems, MAB5457), https://www.rndsystems.com/cn/products/human-mouse-rat-glutathione-peroxidase-4-gpx4-antibody-565320_mab5457<br>tubulin (1:5000; Cell Signaling Technology, #2144), https://www.cellsignal.com/products/primary-antibodies/a-tubulin-antibody/2144<br>vinculin (1:10000; Sigma, V4505), https://www.sigmaaldrich.com/catalog/product/sigma/v4505?lang=en&region=US<br>SLC7All (1:4000; Cell Signaling Technology, #12691) ,https://www.cellsignal.com/products/primary-antibodies/xct-slc7a11-d2m7a-rabbit-mab/12691?site-search-type=Products&N=4294956287&Ntt=xct&fromPage=plp<br>p53 (1:1000; Santa Cruze, sc-126), https://www.scbt.com/p/p53-antibody-do-1?gclid=CjwKCAiAgc-ABhA7EiwAjev-j_vhIk8IBXPiuFK0TiipPp9FQb4LbiUnIgcTy_iZpnnVpOhur3gBRBoCjXAQAvD_BwE

LC3B (1:5000; Cell Signaling Technology,#3868),https://www.cellsignal.com/products/primary-antibodies/lc3b-d11-xp-rabbit-mab/3868
HIF2a (1:1000; Cell Signaling Technology, #7096), https://www.cellsignal.com/products/primary-antibodies/hif-2a-d9e3-rabbit-mab/7096
TSCl (1:1000; Cell Signaling Technology, #6935) https://www.cellsignal.com/products/primary-antibodies/hamartin-tsc1-d43e2-rabbit-mab/6935
phospho-S6K (1:1000; Cell Signaling Technology, #9205), https://www.cellsignal.com/products/primary-antibodies/phospho-p70-s6-kinase-thr389-antibody/9205
S6K (1:1000; Cell Signaling Technology, #9202), https://www.cellsignal.com/products/primary-antibodies/p70-s6-kinase-antibody/9202
P-S6 (1:1000; Cell Signaling Technology, #2215), https://www.cellsignal.com/products/primary-antibodies/phospho-s6-ribosomal-protein-ser240-244-antibody/2215
S6 (1:1000; Cell Signaling Technology, #2217), https://www.cellsignal.com/products/primary-antibodies/s6-ribosomal-protein-5g10-rabbit-mab/2217
P-4EBP1 (1:1000; Cell Signaling Technology, #2855), https://www.cellsignal.com/products/primary-antibodies/phospho-4e-bp1-thr37-46-236b4-rabbit-mab/2855
4EBP1 (1:1000; Cell Signaling Technology, #9644), https://www.cellsignal.com/products/primary-antibodies/4e-bp1-53h11-rabbit-mab/9644
4EBP2 (1:1000; Cell Signaling Technology, #2845), https://www.cellsignal.com/products/primary-antibodies/4e-bp2-antibody/2845
Actin (1:1000; Cell Signaling Technology, #3700), https://www.cellsignal.com/products/primary-antibodies/b-actin-8h10d10-mouse-mab/3700
Rictor (1:1000; Cell Signaling Technology, #2140), https://www.cellsignal.com/products/primary-antibodies/rictor-antibody/2140
puromycin (Developmental Studies Hybridoma Bank, 1:1000, #PMY-2A4), https://dshb.biology.uiowa.edu/PMY-2A4
β-Actin (Sigma-Aldrich, 1:5000, A2228) , https://www.sigmaaldrich.com/catalog/product/sigma/a2228?lang=en&region=US&gclid=CjwKCAiAgc-ABhA7EiwAjev-j27OhD-iMKjl1mlBj4L0QxfmkdPjndzXP3gPcMoWO96HUDBROPxvPRoCzbQQAvD_BwE

Eukaryotic cell lines

Policy information about cell lines

Cell line source(s)
UMRC6 cell line was obtained from Sigma (08090513). HEK-293T (CRL-3216), 786-0 (CRL-1932), ACHN (CRL-1611), NCI-H226 (CRL-5826), H460(HTB-177), NCI-H23(CRL-5800), NCI-H1299(CRL-5803) cell lines were obtained from ATCC.

Authentication
Cell line were not authenticated.

Mycoplasma contamination
All cell lines tested negative for mycoplasma contamination.

Commonly misidentified lines
(See ICLAC register)
No ICLAC cell line was used in this study.

Animals and other organisms

Policy information about studies involving animals; ARRIVE guidelines recommended for reporting animal research

Laboratory animals
NOD scid gamma (NSG) female mice were maintained at a condition of 12-hour light/12-hour dark cycle and temperatures of 65-75°F (18-23°C) with 40-60% humidity.

Wild animals
No wild animals involved in this study.

Field-collected samples
This study didn't involve samples collected from field.

Ethics oversight
All the xenograft model experiments were performed in accordance with a protocol approved by the Institutional Animal Care and Use Committee and Institutional Review Board at The University of Texas MD Anderson Cancer Center. Patient-derived xenograft models were described in the methods. No living human research participants were involved in this study.

Note that full information on the approval of the study protocol must also be provided in the manuscript.

Human research participants

Policy information about studies involving human research participants

Population characteristics
Primary PDX tumors were collected from patients confirmed with the diagnosis of NSCLC. Detailed information on human research participants (age, gender, genotypic information, diagnosis, and treatment categories) is available upon reasonable request.

Recruitment
Patients were recruited based on their own consents. No criteria was applied to determine the recruitment. However, due to limited time to process patient-derived tumor samples, only one patient sample was included in the study. More PDXs originated from different cancer patients should be studied to further evaluate the drug efficacy considering tumor heterogeneity.

Ethics oversight
Patient-derived xenografts (PDXs) were generated in accordance with protocols approved by the Institutional Review Board at The University of Texas MD Anderson Cancer Center. Informed consent was obtained from the patients and the study is

compliant with all relevant ethical regulations regarding research involving human participants. Xenograft experiments were performed in accordance with a protocol approved by the Institutional Animal Care and Use Committee and Institutional Review Board at The University of Texas MD Anderson Cancer Center.

Note that full information on the approval of the study protocol must also be provided in the manuscript.

Flow Cytometry

Plots

Confirm that:

☒ The axis labels state the marker and fluorochrome used (e.g. CD4-FITC).

☒ The axis scales are clearly visible. Include numbers along axes only for bottom left plot of group (a 'group' is an analysis of identical markers).

☒ All plots are contour plots with outliers or pseudocolor plots.

☒ A numerical value for number of cells or percentage (with statistics) is provided.

Methodology

Sample preparation	To measure levels of lipid peroxidation, cells in 12-well plate after treatments were incubated with fresh medium containing 2 µM BODIPY 581/591 Cll dye {lnvitrogen, D3861) for 30 minutes. Then cells were collected and washed once with PBS followed by fluorescence-activated cell sorting (FACS) analysis. Fluorescence in channel 1 in live cells was captured and plotted using FlowJo_ VlO software.
Instrument	Accuri C6 (BD Bioscience)
Software	Using Accuri C6 software to collect data and FlowJo_Vl 0 software to analyze data.
Cell population abundance	At least 2 000-5000 cells were analyzed for each sample.
Gating strategy	Initial cell population gating (FSC-Area Vs FSC-Height) was adopted to make sure only single cells were used for analysis.

☒ Tick this box to confirm that a figure exemplifying the gating strategy is provided in the Supplementary Information.
